# The Effects of Water and Nitrogen Addition on the Allocation Pattern and Stoichiometric Characteristics of C, N, and P in Peanut Seedlings

**DOI:** 10.3390/plants14030353

**Published:** 2025-01-24

**Authors:** Qing Guo, Feifei Qin, Yang Xu, Hao Feng, Guanchu Zhang, Zhimeng Zhang, Yucheng Chi, Hong Ding

**Affiliations:** Shandong Peanut Research Institute, Shandong Academy of Agricultural Sciences, Qingdao 266100, China; jone007@126.com (Q.G.); jialing_300@163.com (F.Q.); xy52120092661@163.com (Y.X.); ben0917@163.com (H.F.); guanchuzhang@126.com (G.Z.); qinhdao@126.com (Z.Z.)

**Keywords:** peanut seedlings, nitrogen and water addition, differentiated peanut organs, nitrogen fertilization, stoichiometric characteristics

## Abstract

Water and fertilizer application strategies seriously affect the healthy growth of peanuts. The stoichiometric ratio can directly reflect the elemental requirements for crop growth, which are very important for improving fertilizer utilization efficiency. In order to investigate the response of C (carbon), N (nitrogen), and P (phosphorus) allocation pattern and stoichiometric characteristics in peanut seedlings to water and nitrogen addition, we designed a greenhouse pot experiment which had different water treatments (W1, 75–80% field capacity; W2, 45–50% field capacity) and nitrogen addition treatments (N0, 0 kg hm^−2^; N1, 90 kg hm^−2^; N2, 180 kg hm^−2^). The distribution and content changes in C, N, and P in different organs were measured and analyzed by ecological stoichiometry. The results showed that drought stress significantly increased the N or P content of different organs. The average N/P value of peanut roots treated with W2 decreased by 13.02% compared to W1. Restoring irrigation relieved this stress while reducing the C/N and C/P of roots, stems, and leaves, as well as the N/P of roots and stems. The water treatment after rehydration showed significant differences in the C/N, C/P, and N/P ratios of peanut roots. The average values of the C/N, C/P, and N/P ratios of peanut roots in W2 treatment were reduced by 13.54%, 28.66%, and 16.34%, respectively, compared to W1 treatment. On the other hand, nitrogen application significantly increased the N content of stems and leaves, while reducing C/N. On the contrary, it significantly reduced the P content of roots and stems, and increased the N/P ratio of roots, stems, and leaves. Overall, there is a significant interaction between water and nitrogen treatment on the stoichiometric characteristics of C, N, and P in different organs, with water treatment playing a dominant role. In terms of nutrient distribution in organs, the average N content in leaves is the highest. The coefficient of variation (CV) of P content is greater than that of C and N content. The CV of N content, P content, and C/N and N/P ratios of the stem are all greater than those of the roots or leaves, while the stems are more sensitive to water and nitrogen conditions. And the N and P content of roots, stems, and leaves were positively correlated. Meanwhile, peanut seedlings have the phenomenon of ionic synergism that occurs between nitrogen and phosphorus ions. In summary, studying the stoichiometric ratios can reflect the water and fertilizer demand status of peanuts, thereby better improving water and fertilizer utilization efficiency.

## 1. Introduction

Drought occurs more and more frequently with the intensification of global climate change, which poses a serious threat to global agricultural production and the ecological environment. Drought disrupts vegetation and the hydrological cycle, leading to soil infertility and the loss of biodiversity, threatening the integrity and stability of ecosystems [1]. Li et al. [2] reported that drought stress inhibited the activity of enzymes related to element cycling and transformation in soil; limited the processes of soil weathering, organic matter generation, and mineralization; and slowed down the release of nutrient elements. Meanwhile, it reduced the activity of soil microorganisms, content of available nutrients, and utilization and accumulation of nutrients by plants, ultimately affecting nutrient allocation [3].

Water and fertilizer management strategies are key factors affecting crop yield. Reasonable water and fertilizer management can not only increase yield, but also reduce environmental pollution [4]. Nitrogen and phosphorus fertilizers, as the main fertilizers for peanut growth, have been widely reported in terms of their concentration and usage methods. However, at present, the utilization efficiency of N and P fertilizers is still not very high, which not only causes waste but also exacerbates environmental pollution [5]. Therefore, strengthening the rationality of peanut water and fertilizer management has important value and significance [6]. Ecological stoichiometry is the science that studies the balance of chemical elements in ecosystems. N and P are the most fundamental nutrients for plant growth, whose ecological stoichiometric characteristics reflect the internal stability of plant and the response to changes in the external environment, including the assessment of plant growth rate and nutrient utilization [7,8]. Wang et al. [9] suggested that plants could adapt to changes in the external environment by actively adjusting nutrient requirements and the relative abundance of nutrient elements. Liu et al. [10] found that the optimal stoichiometric ratios of chemical elements were different due to plants’ different response pathways and metabolic patterns to various external stresses. Therefore, studying the changes in C, N, and P content and ecological stoichiometric ratios during the growth of individual plants under different environmental conditions is more targeted in revealing the interactions between the plant and the environment.

As an important oil crop and economic crop, strengthening water and fertilizer management for peanuts is crucial for ensuring food and oil security. Numerous studies have shown that suitable water and nitrogen conditions can promote the growth and development of peanuts, improving yield and quality. For example, Guo et al. [11] reported that proper fertilization and irrigation can ensure peanut yield without causing environmental pollution. On the contrary, adverse conditions have a negative impact on growth and development, leading to a decrease in yield and poor quality. For example, Zhang et al. [12] reported that excessive application of nitrogen fertilizer can affect the growth of peanuts by affecting soil fungal structure [13]. Therefore, further strengthening agricultural water and fertilizer management strategies is crucial for increasing peanut yields. C, N, and P are the most important biogenic elements in peanuts, involved in basic life processes such as energy transfer, information expression, and genetic variation [8]. C is an important substrate and energy substance for plant growth, involved in various physiological and biochemical processes in plants, while N and P are important nutrients and key elements for plant cell composition and metabolism [14]. The ecological stoichiometry of these elements directly reflects the proportion of nutrients required for plant growth and the current growth status [15,16]. Therefore, studying the C, N, and P content and stoichiometric ratio of plants can help reveal their growth, development, and nutrient absorption and utilization efficiency, thereby better improving fertilizer utilization efficiency [17].

Currently, most research focuses on the impact of water and N fertilizer application on the physiological functions of peanut. There are few reports on the influence of water and nitrogen application on the stoichiometric characteristics of C, N, and P in peanuts. Therefore, the purpose of the study is to investigate the effects of different water and nitrogen addition treatments on the C, N, and P content and ecological stoichiometry of peanuts. The research objectives are (1) to explore the dynamic changes in C, N, and P content in different organs of peanuts, and (2) to explore changes in peanut stoichiometry under different water and nitrogen treatments. Finally, we hope that this research can provide guidance for better irrigation and fertilization of peanuts.

## 2. Results

### 2.1. Changes in C, N, and P Content and Stoichiometric Ratios of Peanut Roots

Water treatment had a significant effect on the C and P content of the roots. The average content of C and P in peanut roots under W2 increased by 6.04% and 13.76% compared with W1, respectively (Table 1). There were extremely significant differences in the N and P content of the roots under water treatment. The average N and P content in the roots under W2 increased by 15.31% and 35.46% compared with W1. Nitrogen treatment exhibited significant differences in the C and P content of the roots under drought stress, with the average values showing a trend of N1 > N0 > N2. Nitrogen treatment had extremely significant differences in N and P content after rewatering. The N content of the roots increased with the increase in the nitrogen application rate, with the highest root N content observed in W2N2 (18.11 g kg^−1^). There was a significant interaction between nitrogen treatment and water treatment on root N content. The average P content in the roots was highest in the N0 group, while the maximum root P content was found under W2N0 (2.81 g kg^−1^).

Water treatment showed significant differences in N/P in the roots under water stress, with the average N/P in the roots under W2 decreased by 13.02% compared to W1. The average values of C/N, C/P, and N/P in the roots under W2 significantly decreased by 13.54%, 28.66%, and 16.34% compared with W1 after rewatering, respectively (Table 2). Nitrogen treatment made a significant difference to the N/P of the roots under water stress, with N2 having the highest average of N/P. There were extremely significant differences in C/N, C/P, and N/P in the roots among the different nitrogen treatments after rewatering. The C/N of the roots decreased with an increasing nitrogen application rate under different water treatments, while the N/P of the roots increased with an increasing nitrogen application rate under different water treatments. There was a significant interaction effect on C/P between water and nitrogen treatment.

### 2.2. Changes in C, N, and P Content and Stoichiometric Ratios of Peanut Stems

The N and P content in peanut stems under W2 were significantly higher than those of W1 (Table 3). Nitrogen treatment exhibited significant differences in the N and P content of the stems before and after rewatering. Specifically, the N content of the stems increased with the increment of the nitrogen application rate, while the P content of the stems decreased with the increase in nitrogen application. There was a significant interaction on the N content in the stems between water and nitrogen treatment before rewatering, with the highest N content observed in W2N1 (21.17 g kg^−1^).

The average C/N and C/P in the stems under W2 decreased by 20.44% and 11.07% compared with W1, an extremely significant difference (Table 4). The average C/N, C/P, and N/P in the stems under W2 decreased significantly by 11.19%, 20.14%, and 14.46% compared with W1, respectively. There were significant differences in C/N, C/P, and N/P in the stems before and after rewatering. The C/N of the stems decreased with the increase in fertilizer application rate, while the C/P and N/P of the stems increased with the incrementing of the nitrogen application rate.

### 2.3. Changes in C, N, and P Content and Stoichiometric Ratios of Peanut Leaves

There was an extremely significant difference in the N content of leaves with different water treatments, and the average N content in peanut leaves under W2 significantly increased by 3.28% compared with W1 (Table 5). Water treatment had significant effects on the C, N, and P content in the leaves after rewatering. Specifically, the average content of C, N, and P in the leaves under W2 increased by 2.44%, 23.17%, and 24.22%, respectively, compared with W1. There were extremely significant differences in the N content of peanut leaves before and after rewatering among different nitrogen treatments. The N content of the leaves increased with the increase in the nitrogen application rate. Nitrogen treatment made significant difference to the P content of the leaves and their interaction with water treatment after rewatering. The P content in the leaves under W2N1 achieved the highest value of 2.95 g kg^−1^.

Water treatment had a significant effect on the C/N of peanut leaves under water stress, with the average of C/N in the leaves under W2 decreased by 4.44% compared with W1 (Table 6). After rewatering, water treatment exhibited significant differences in both the C/N and C/P of the leaves, with the average C/N and C/P under W2 decreased by 17.25% and 17.22%, respectively, compared with W1. There were significant differences in the C/N and N/P of the leaves under water stress. The C/N of the leaves decreased as the amount of nitrogen application increased, while the N/P of the leaves increased with increasing nitrogen application. There was a significant interaction between water treatment and nitrogen treatment on leaf C/N under water stress, with W2N2 exhibiting the lowest C/N value in the leaves. Nitrogen treatment showed extremely significant differences in the C/N, C/P, and N/P of peanut leaves after rewatering. The C/N and C/P of the leaves decreased with increasing nitrogen application, while the N/P of the leaves increased with increasing nitrogen application.

### 2.4. Distribution Characteristics of C, N, and P Content and Stoichiometric Ratios in Roots, Stems, and Leaves

The variation range of C content was the largest, spanning from 5.69 to 525.21 g kg^−1^ (Table 7). The C content in the leaves was higher than that in the roots and stems. The range of N content varied from 2.44 to 37.45 g kg^−1^, with the highest N content found in the leaves, followed by the roots. The variation range of P content was the smallest, ranging from 0.33 to 19.5 g kg^−1^, with similar P content observed in various organs. The C/N was the highest in the stems, the C/P was the highest in the roots, and the N/P was the highest in the leaves (Table 7). The variation coefficients of C content in different organs were similar. The CV of the P content was notably larger than that of the C and N contents. The CVs of N and P content, C/N, and N/P in the stems were greater than those in the roots or leaves.

### 2.5. Correlation Among C, N, and P Contents and Stoichiometric Ratios in Peanut Roots, Stems, and Leaves

**Figure 1:**
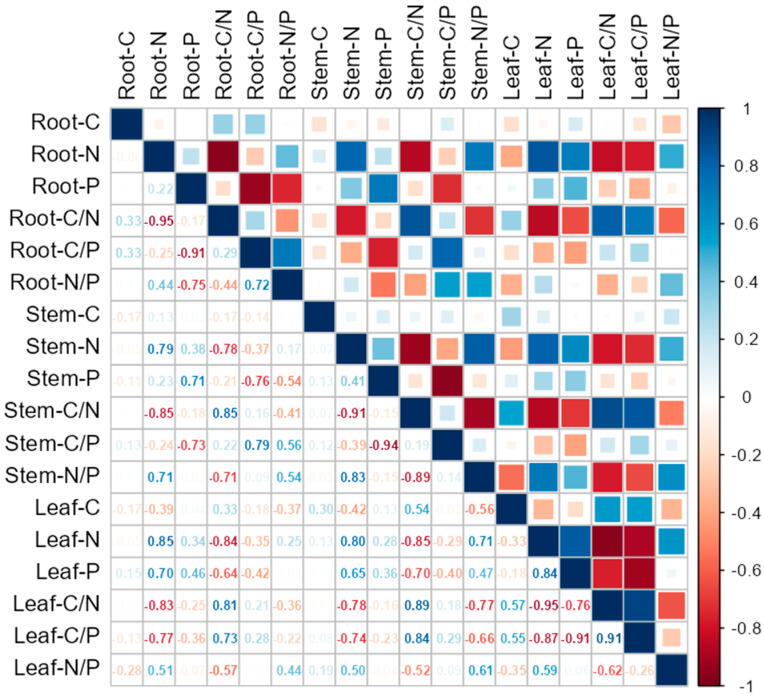
The correlations among C, N, and P content and stoichiometric ratios in peanut roots, stems, and leaves. The color soil column represents correlation; red represents a positive correlation and blue represents a negative correlation.

There was a very significant positive correlation between N content in the roots and that in the stems and leaves, with a greater correlation strength with leaves (Figure 1). The content of N in roots, stems, and leaves was negatively correlated with C/N in all organs, and positively correlated with N/P in stems and leaves. There was a significant positive correlation between the N content of the stems and leaves and the corresponding P content, and a significant negative correlation between the N content of stems and the C/P of roots, stems, and leaves. The P content in the roots exhibited a very significant positive correlation with the P content in the stems and leaves, with a higher correlation strength with stems. The P content in the roots, stems, and leaves showed a very significant negative correlation with C/P in different organs. The P content in the leaves was positively correlated with N content and negatively correlated with C/N in different organs.

## 3. Discussion

### 3.1. Effects of Water and Nitrogen Addition on C, N, and P Content of Peanut Roots, Stems, and Leaves

Water and fertilizer management, as key factors in plant growth, seriously affect plant growth and yield. The W2 treatment significantly increased the C content of peanut roots under water stress, while there was no significant difference in the C content of roots after rewatering (Table 1). Zhang et al. [18] reported that plants allocated more photosynthetic products to their roots by adjusting nutrient allocation strategies under drought stress, so as to enhance the water absorption capacity and stress resistance of roots. This process not only promoted root growth and expansion, but also indirectly led to an increase in the C content of roots. The N and P content of roots under W2 were significantly increased after rewatering, indicating a rapid recovery of growth and metabolic balance in the roots (Table 1). Appropriate nitrogen treatment enhanced root vitality, improved root water absorption ability, promoted the growth and development of peanut roots, and thus increased root C content under drought stress [19]. The photosynthesis capacity of the plants increased as rehydration proceeded, which helped to accumulate more carbon and redistribute it in the plant.

Water stress had no significant effect on the N content of peanut roots (Table 1). However, the average N content of peanut roots under W2 increased significantly after rewatering, indicating that rehydration promoted the absorption of N by the roots. The recovery of water improved the soil environment, promoted the growth and metabolic activities of the roots, and enhanced the ability of the roots to absorb nutrients such as N [20,21]. Hu et al. [22] suggested that rehydration promotes the division and elongation of root cells and increases the absorption area of roots. Additionally, the enhancement of root metabolic activities also improves the expression and activity of transporters related to N absorption, thereby facilitating the transport and accumulation of N in the roots [23]. The N content of the roots increased significantly after rewatering which demonstrated that the rapid growth of the roots after rehydration promoted the absorption of N from soil [24].

However, the P content of the roots in W2 decreased after rewatering compared with that under water stress (Table 1). P plays a crucial role in the metabolism of carbohydrates, fats, and proteins [25]. The peanut roots enhanced their physiological function and stress resistance by increasing the absorption of P under drought conditions. The plant redistributed more P to its aboveground parts to support its rapid growth and developmental needs after rewatering, resulting in a relative reduction in P content in the roots [26].

W2 significantly increased the N and P contents of peanut stems before and after rewatering (Table 3). Sun H. [27] suggested that drought stress triggers a series of physiological responses in peanut plants, such as the synthesis of osmoregulatory substances (proline, betaine, stress protein and so on), which enhances the absorption and storage of N and P. N is a fundamental element that constitutes key biomolecules such as proteins, nucleic acids, and chlorophyll in plants. The plants were able to take up more N and distribute it to various organs, including the stem, when the amount of nitrogen fertilizer applied increased. There was a competitive relationship between the absorption and utilization of N and P in the soil, which inhibited the absorption of P [28].

Water treatment made no significant difference to the C content of the leaves under water stress, but significantly increased the C content of the leaves after rewatering (Table 5). Plants treated with drought stress developed rapidly after rehydration; the water content of the leaves increased and the stomata reopened which helped the plants conduct photosynthesis more efficiently [29]. W2 significantly increased the N content of the leaves under water stress and after rewatering, with a notable 23.17% increase in the N content of the leaves under W2. Rehydration provided the necessary water conditions for the plants and promoted the absorption and transport of N from the soil by the roots. The metabolic activity of plants was enhanced, and the ability of N assimilation and utilization was improved after rehydration [30]. Furthermore, rewatering also stimulated some physiological mechanisms in the plants, such as antioxidant enzymes and antioxidant substances, that promoted the reabsorption and reuse of N [31].

The N content of the leaves significantly increased with the increase in nitrogen fertilizer application (Table 5), indicating that the leaves were more sensitive to nitrogen fertilizer. There was significant interaction between water treatment and nitrogen treatment on the N content of the stems under water stress. However, drought stress inhibited this trend, impeding the absorption of N nutrients. Xiu et al. [32] found that the roots of peanut plants reduced N absorption due to insufficient water under drought conditions, and the increased application of nitrogen fertilizer also increased the resistance of the roots to soil water absorption.

Water treatment had no significant effect on the P content of peanut leaves under water stress, but significantly increased the P content of the leaves under W2 after rewatering (Table 5). Rewatering provides the necessary water environment for plant roots, allowing them to more efficiently absorb water from the soil and the minerals dissolved in the water. After rewatering, the physiological metabolism of the plants gradually recovered, and the plants redistributed nutrient resources, including transporting more P to their photosynthetic active parts such as the leaves [33]. Nitrogen treatment had no significant influence on the P content of peanut leaves under water stress, but significantly increased the P content of leaves and exhibited a significant interaction with water treatment after rewatering. This indicated that water and nitrogen treatments had a synergistic effect in promoting plants’ uptake and utilization of P. Yan et al. [34] believe that water and nitrogen treatment not only directly affect the absorption of P by plants, but also indirectly promote the release and availability of P by improving the soil environment such as increasing soil pH and soil microbial activity.

### 3.2. Effects of Water and Nitrogen Addition on Stoichiometric Ratios of Peanut Roots, Stems, and Leaves

Water treatment made no significant difference to the C/N and C/P of peanut roots under water stress (Table 2). The water status changed significantly after rehydration, which stimulated the rapid resumption of physiological activities and accelerated metabolic processes in the seedling roots [35]. This rapid physiological response facilitated the absorption of N and P contents by the roots (Table 1). There was significant interaction between water and nitrogen treatment on the C/P of the roots after rewatering. Li et al. [36] reported that there was a balance between the promoting effect of nitrogen fertilizer and the inhibiting effect of water stress on plant growth. The promotion effect of nitrogen fertilizer was dominant under normal water supply conditions. However, the plants needed to adjust their metabolism to adapt to the drought stress, which may have resulted in a different trend in C/P changes. On the other hand, peanuts, as a leguminous plant, have strong N fixation ability in their roots, which is also one of the main reasons affecting the root C/N ratio, including root nodule N fixation, etc. Yang et al. [37] also found the same result in alfalfa and attributed it to the N fixation ability of their roots.

The C/P in stems increased with the increase in N application before and after rewatering (Table 4). Drought affected the physicochemical properties of soil, microbial activities, and the dissolution and transformation of P, which reduced the availability of P in the soil [38]. The plants tended to absorb more N after rehydration in order to quickly recover growth and compensate for the nutrient deficiency during the drought. The increase in N promoted the fixation and metabolism of C in plants, which may lead to the relative increase in the content of C. We also found that the C/N of leaves in W2 decreased significantly compared with W1 before and after rewatering, respectively (Table 6). The C/N and C/P of the leaves represent the ability to absorb nutrients and assimilate carbon, reflecting the productivity achievable per unit nutrient supply and the utilization efficiency of nutrients by the plants, which has important ecological significance [39]. The plants reduced carbon fixation through decreased photosynthesis, and enhanced drought resistance by increasing N absorption or translocation under drought stress [40]. N translocation and utilization became more efficient after rewatering, as physiological activities resumed and metabolic rates increased, which led to an increased accumulation of N in the leaves and the further decreasing of C/N.

There were significant differences in the C/N and N/P of leaves under nitrogen treatment before and after rewatering (Table 6). N and P are essential macronutrients for plant growth, and N/P can serve as an indicator of the nutrient supply status of the environment for plant growth [7]. Previous studies have indicated that plants were limited by N when N/P < 14, by P when N/P > 16, and co-limited by both N and P when N/P fell between 14 and 16 [41]. The N/P < 14 in roots, stems, and leaves indicated that plant growth was restricted by N under different water and nitrogen conditions.

### 3.3. Distribution Strategy of C, N, and P in Peanut Roots, Stems, and Leaves

The average N content in leaves was the highest due to photosynthesis requiring a large amount of N for synthetase and pigments [42]. The coefficient of variation for P content in roots, stems, and leaves was significantly larger than that of the C and N contents, indicating that P was more sensitive to water and nitrogen conditions. The coefficients of variation for N content, P content, C/N, and N/P in the stems were greater than those in the roots or leaves (Table 7). The stem is an important hub for the transport of nutrients and water within the plant as the connecting part between the roots and leaves. Shu et al. [43] reported that nutrient allocation was influenced by competition and balance between roots and leaves and the physiological activities and metabolic processes in the stems were more complex and variable, with greater fluctuation.

### 3.4. Correlation Among C, N, and P Contents in Peanut Roots, Stems, and Leaves

C, N, and P, as essential elements for plant growth, have important significance in characterizing the growth status of plants in terms of their different contents and proportions. The stoichiometric ratios of C/N, C/P, and N/P in alfalfa leaves have been reported to be greatly influenced by planting years, but there was no unidirectional change trend with the increase in planting years [44]. Lin et al. [45] reports that the stoichiometric ratio directly reflects the nutrient utilization efficiency of plants. In our experimental results, the N content in the roots was positively correlated with that in the stems and leaves, and the correlation was higher with the leaves than with the stems (Figure 1). This indicated that leaves have a higher demand for N, and the N absorbed by the roots was preferentially allocated to leaves [46]. The N contents of the stems and leaves were positively correlated with the corresponding P content, indicating that there was a synergistic effect on the absorption and utilization of N and P by the plants. The N content in the stems exhibited a significant negative correlation with C/P in the roots, stems, and leaves, which means that the plants achieved precise regulation of growth rate and energy metabolism by regulating the absorption and distribution of N and the metabolism and utilization of C and P during growth and development. This regulatory mechanism helped the plants adapt to different growth environments and cope with various biological stresses [47]. The P content in the roots was significantly positively correlated with that in the stems and leaves, with the correlation coefficient being higher with the stems than with the leaves. This suggested that stems were more sensitive to demands and responses to P, and played a more crucial role in the transport and allocation of P. The P content in the leaves was positively correlated with N content in the roots and stems, and negatively correlated with C/N in the roots and stems, reflecting the synergistic effect of P and N in the plants, which helped to maintain the normal physiological functions of the plants in different water and nitrogen environments [48].

## 4. Materials and Methods

### 4.1. Experimental Design

The experiment was conducted in pot culture with rainproof shelter at Laixi Experimental Station of Shandong Peanut Research Institute (E: 120°53′, W: 36°86′) in 2021. Each pot contained 20 kg of soil with a diameter of 32 cm and a height of 26 cm. The basic physical and chemical properties of the experimental soil are shown in Table 8. The experiment was designed in a randomized block design with two water treatments and three nitrogen fertilizer treatments. Water treatment consisted of W1 (normal water supply during the seedling stage, 75–80% of field capacity) and W2 (drought stress during the seedling stage, 45–50% of field capacity) [49]. The soil water content was measured by electronic weighing. Specific field capacity was controlled by water control starting from 30 days after sowing, and the drought stress lasted for 20 days. Fields were rewatered immediately after the end of drought stress. Nitrogen treatments included N0 (0 kg hm^−2^, no nitrogen), N1 (90 kg hm^−2^, moderate nitrogen) and N2 (180 kg hm^−2^, high nitrogen) [50,51]. Nitrogen was applied in the form of urea (N content 46%), along with the application of calcium dihydrogen phosphate (450 kg hm^−2^) and potassium sulfate (300 kg hm^−2^). N0 treatment received the same P and K fertilizer. All fertilizers were applied as base fertilizers, and the other field managements were the same as the general high-yield fields. Normal water supply was maintained during sowing to ensure the quality of peanut emergence, with 4 seeds planted in each pot. Thinning was carried out after emergence to retain 2 uniform seedlings in each pot. There were 6 repetitions in each treatment and 36 pots in total. Six plants from each treatment were sampled at the end of 20 days of water stress and 20 days after rewatering in the seedling stage.

### 4.2. Sampling and Measurement

Peanut variety “Huayu 25” (Shandong Peanut Research Institute, Qingdao, China) was tested in the experiment.

We wiped off the surface moisture after rinsing the peanut plants thoroughly. These plants were separated into roots, stems, and leaves and were oven-dried at 105 °C for 30 min. Then, they were dried at 65 °C to a constant weight and the final weight was measured. Each part of the samples was ground through a 100-mesh screen after drying.

The total C and N content of the samples were determined by a C/N Elemental Analyzer (2400II CHNS/O Elemental Analyzer, Perkin-Elmer, Waltham, MA, USA). Total P content was found by the concentrated sulfuric acid–perchloric acid digestion method and determined by the molybdenum–antimony yellow colorimetric method [9].

### 4.3. Statistical Analysis

Data processing was conducted in Excel 2016 (Microsoft Corp., Redmond, NM, USA), and two-way analysis of variance (ANOVA) was performed to analyze C, N, and P content and stoichiometric ratios between water treatment and nitrogen addition treatment in R 4.0.2. Multiple comparisons were made using least significant difference (LSD) tests at *p* < 0.05. The relationship between C, N, and P content and stoichiometric ratios in each organ was analyzed by a Pearson correlation analysis.

## 5. Conclusions

Drought stress significantly increased N or P contents in different organs, while water addition significantly improved both N and P contents in all the organs, and reduced the C/N and C/P of the roots, stems, and leaves, and the N/P of roots and stems. Nitrogen addition significantly increased N content and decreased the C/N of stems and leaves. Meanwhile, it notably reduced the P content of the roots and stems, and improved the N/P of the roots, stems, and leaves. Water and nitrogen treatment have significant interactions with the stoichiometric characteristics of C, N, and P in different organs, in which water treatment played a dominant role. The mean N content in the leaves was the highest. The coefficient of variation (CV) of P content was greater than that of C and N content. The CVs of N content, P content, C/N, and N/P of the stems were greater than those of the roots or leaves. The stem was more sensitive to water and nitrogen conditions. The contents of N and P in different organs were positively correlated. Peanut seedlings tended to absorb N, with their leaves having a higher demand for N and their stems having a greater need for P. There was a synergistic effect on the absorption and utilization of N and P by peanut seedlings. In the future, more in-depth correlation analysis and research are needed in field experiments to provide more guidance for agricultural production.

## Figures and Tables

**Table 1:** Effects of water and nitrogen addition on C, N, and P content of peanut roots under water stress and rewatering at seedling stage.

Water (W)	Nitrogen (N)	Water Stress			Rewatering		
		C	N	P	C	N	P
W1	N0	426.06 ± 27.25 a	18.00 ± 0.32 a	2.27 ± 0.09 a	442.88 ± 18.83 a	11.40 ± 0.13 c	2.26 ± 0.14 a
	N1	434.47 ± 10.59 a	17.20 ± 1.37 a	2.81 ± 0.58 a	446.64 ± 4.00 a	13.41 ± 0.72 b	1.81 ± 0.15 b
	N2	407.46 ± 8.43 a	16.84 ± 1.55 a	2.26 ± 0.31 a	465.45 ± 20.22 a	15.17 ± 0.41 a	1.57 ± 0.09 b
	Mean	422.66	17.35	2.45	451.66	13.33	1.88
W2	N0	453.06 ± 16.67 ab	16.68 ± 0.84 a	2.99 ± 0.06 a	426.72 ± 12.85 a	12.01 ± 0.88 c	2.81 ± 0.48 a
	N1	470.32 ± 15.77 a	17.58 ± 0.69 a	2.91 ± 0.08 a	469.44 ± 23.58 a	15.98 ± 0.87 b	2.33 ± 0.09 a
	N2	421.19 ± 24.24 b	17.82 ± 2.03 a	2.45 ± 0.02 b	430.48 ± 26.23 a	18.11 ± 1.00 a	2.50 ± 0.25 a
	Mean	448.19	17.36	2.78	442.21	15.37	2.55
Source of variation							
Water (W)		*	ns	*	ns	**	**
Nitrogen (N)		*	ns	*	ns	**	**
W * N		ns	ns	ns	ns	*	ns

Note: Values within a column followed by the same letters do not differ significantly (*p* ≤ 0.05) according to LSD tests. W, water addition. N, nitrogen addition. ns, not significant. * significant at *p* < 0.05. ** significant at *p* < 0.01.

**Table 2:** Effects of water and nitrogen addition on C/N, C/P, and N/P of peanut roots under water stress and rewatering at seedling stage.

Water (W)	Nitrogen (N)	Water Stress				Rewatering		
		C/N	C/P	N/P		C/N	C/P	N/P
W1	N0	188.14 ± 19.85 a	7.93 ± 0.28 a		38.85 ± 1.24 a	196.30 ± 12.40 c	5.05 ± 0.29 c	23.69 ± 1.7 a
	N1	158.76 ± 33.27 a	6.22 ± 0.85 a		33.36 ± 1.91 b	248.24 ± 21.44 b	7.43 ± 0.30 b	25.36 ± 1.89 a
	N2	181.88 ± 22.35 a	7.58 ± 1.58 a		30.67 ± 0.51 c	296.49 ± 12.57 a	9.67 ± 0.42 a	24.36 ± 2.68 a
	Mean	176.26	7.24		34.29	247.01	7.38	24.47
W2	N0	151.52 ± 8.03 a	5.58 ± 0.32 b		35.68 ± 3.42 a	153.83 ± 19.83 b	4.37 ± 0.95 b	27.22 ± 1.95 a
	N1	161.54 ± 3.10 a	6.05 ± 0.40 b		29.48 ± 3.03 b	201.97 ± 15.95 a	6.87 ± 0.37 a	26.80 ± 1.85 a
	N2	171.72 ± 8.53 a	7.27 ± 0.79 a		23.79 ± 1.26 c	172.86 ± 7.96 ab	7.29 ± 0.71 a	23.76 ± 1.86 a
	Mean	188.14 ± 19.85 a	7.93 ± 0.28 a		38.85 ± 1.24 a	196.30 ± 12.40 c	5.05 ± 0.29 c	23.69 ± 1.7 a
Source of variation								
Water (W)		ns	ns	*		**	**	**
Nitrogen (N)		ns	ns	*		**	**	**
W * N		ns	ns	ns		ns	**	*

Note: Values within a column followed by the same letters do not differ significantly (*p* ≤ 0.05) according to LSD tests. W, water addition. N, nitrogen addition. ns, not significant. * significant at *p* < 0.05. ** significant at *p* < 0.01.

**Table 3:** Effects of water and nitrogen addition on C, N, and P content of peanut stems under water stress and rewatering at seedling stage.

Water (W)	Nitrogen (N)	Water Stress			Rewatering		
		C	N	P	C	N	P
W1	N0	434.69 ± 33.51 a	13.90 ± 0.27 b	2.76 ± 0.18 a	439.78 ± 21.68 a	5.57 ± 0.39 c	2.61 ± 0.53 a
	N1	428.49 ± 17.60 a	16.19 ± 1.47 a	2.54 ± 0.20 ab	432.03 ± 25.24 a	9.30 ± 1.57 b	1.76 ± 0.28 b
	N2	435.35 ± 29.16 a	17.91 ± 0.53 a	2.40 ± 0.09 b	421.41 ± 31.69 a	11.43 ± 0.82 a	1.60 ± 0.13 b
	Mean	432.84	16.00	2.57	431.07	8.77	1.99
W2	N0	415.21 ± 23.67 a	18.93 ± 0.60 b	2.96 ± 0.13 a	434.25 ± 10.97 a	6.54 ± 0.90 c	2.71 ± 0.40 a
	N1	453.94 ± 42.32 a	21.17 ± 0.60 a	3.01 ± 0.20 a	461.25 ± 29.55 a	10.98 ± 0.26 b	2.35 ± 0.10 a
	N2	455.71 ± 27.10 a	20.70 ± 0.51 a	2.87 ± 0.38 a	453.50 ± 59.81 a	12.88 ± 0.73 a	2.40 ± 0.10 a
	Mean	441.62	20.27	2.95	449.67	10.13	2.49
Source of variation							
Water (W)		ns	**	**	ns	**	**
Nitrogen (N)		ns	**	*	ns	**	**
W * N		ns	*	ns	ns	ns	ns

Note: Values within a column followed by the same letters do not differ significantly (*p* ≤ 0.05) according to LSD tests. W, water addition. N, nitrogen addition. ns, not significant. * significant at *p* < 0.05. ** significant at *p* < 0.01.

**Table 4:** Effects of water and nitrogen addition on C/N, C/P, and N/P of peanut stems under water stress and rewatering at seedling stage.

Water (W)	Nitrogen (N)	Water Stress			Rewatering		
		C/N	C/P	N/P	C/N	C/P	N/P
W1	N0	31.31 ± 2.89 a	157.71 ± 2.92 b	5.06 ± 0.38 b	79.00 ± 1.97 a	174.25 ± 42.78 b	2.21 ± 0.58 c
	N1	26.58 ± 1.98 b	169.06 ± 10.09 ab	6.40 ± 0.88 a	47.16 ± 6.74 b	249.01 ± 36.81 a	5.28 ± 0.22 b
	N2	24.30 ± 1.23 b	181.45 ± 5.63 a	7.47 ± 0.22 a	36.87 ± 1.49 c	264.10 ± 5.42 a	7.17 ± 0.17 a
	Mean	27.40	169.41	6.31	54.34	229.12	4.89
W2	N0	21.93 ± 0.99 a	140.41 ± 13.06 a	6.39 ± 0.33 a	67.35 ± 10.85 a	162.25 ± 20.30 a	2.48 ± 0.65 b
	N1	21.43 ± 1.71 a	151.53 ± 21.73 a	7.05 ± 0.46 a	42.04 ± 3.06 b	196.98 ± 16.88 a	4.68 ± 0.08 a
	N2	22.03 ± 1.45 a	160.02 ± 12.09 a	7.31 ± 1.00 a	35.40 ± 6.10 b	189.69 ± 27.87 a	5.38 ± 0.49 a
	Mean	21.80	150.65	6.92	48.26	182.97	4.18
Source of variation							
Water (W)		**	**	ns	*	**	**
Nitrogen (N)		*	*	**	**	**	**
W * N		*	ns	ns	ns	ns	**

Note: Values within a column followed by the same letters do not differ significantly (*p* ≤ 0.05) according to LSD tests. W, water addition. N, nitrogen addition. ns, not significant. * significant at *p* < 0.05. ** significant at *p* < 0.01.

**Table 5:** Effects of water and nitrogen addition on C, N, and P content of peanut leaves under water stress and rewatering at seedling stage.

Water (W)	Nitrogen (N)	Water Stress			Rewatering		
		C	N	P	C	N	P
W1	N0	470.77 ± 4.15 a	28.03 ± 1.00 b	2.69 ± 0.13 a	506.62 ± 9.22 a	18.81 ± 1.36 b	1.96 ± 0.06 b
	N1	454.17 ± 4.35 a	29.78 ± 0.47 a	2.79 ± 0.17 a	461.7 ± 29.82 ab	22.50 ± 3.57 ab	2.08 ± 0.12 b
	N2	464.35 ± 0.38 a	30.51 ± 0.30 a	2.58 ± 0.13 a	447.30 ± 26.08 b	26.24 ± 1.18 a	2.36 ± 0.18 a
	Mean	463.10	29.44	2.69	471.87	22.52	2.13
W2	N0	445.09 ± 30.21 a	29.71 ± 0.48 b	2.71 ± 0.04 a	495.55 ± 19.70 a	22.47 ± 2.10 b	2.34 ± 0.16 b
	N1	477.85 ± 19.32 a	30.23 ± 0.51 b	2.90 ± 0.30 a	477.85 ± 41.10 a	30.01 ± 0.62 a	2.95 ± 0.24 a
	N2	450.62 ± 30.78 a	31.28 ± 0.55 a	2.68 ± 0.10 a	476.74 ± 39.41 a	30.72 ± 0.34 a	2.66 ± 0.10 ab
	Mean	457.85	30.41	2.76	483.38	27.73	2.65
Source of variation							
Water (W)		ns	**	ns	*	**	**
Nitrogen (N)		ns	**	ns	ns	**	**
W * N		ns	ns	ns	ns	ns	*

Note: Values within a column followed by the same letters do not differ significantly (*p* ≤ 0.05) according to LSD tests. W, water addition. N, nitrogen addition. ns, not significant. * significant at *p* < 0.05. ** significant at *p* < 0.01.

**Table 6:** Effects of water and nitrogen addition on C/N, C/P, and N/P of peanut leaves under water stress and rewatering at seedling stage.

Water (W)	Nitrogen (N)	Water Stress			Rewatering		
		C/N	C/P	N/P	C/N	C/P	N/P
W1	N0	16.81 ± 0.49 a	174.97 ± 7.49 a	10.41 ± 0.17 b	27.04 ± 2.40 a	259.30 ± 12.82 a	9.61 ± 0.42 a
	N1	15.25 ± 0.22 b	162.96 ± 9.91 a	10.68 ± 0.50 b	20.79 ± 2.71 b	221.87 ± 12.47 b	10.76 ± 1.13 a
	N2	15.22 ± 0.14 b	180.56 ± 9.02 a	11.86 ± 0.54 a	17.04 ± 0.33 b	189.96 ± 6.82 c	11.15 ± 0.60 a
	Mean	15.76	172.83	10.98	21.62	223.71	10.51
W2	N0	14.98 ± 0.95 a	164.48 ± 9.49 a	10.98 ± 0.27 ab	22.21 ± 2.56 a	212.50 ± 19.75 a	9.65 ± 1.33 b
	N1	15.80 ± 0.46 a	165.55 ± 11.17 a	10.49 ± 0.87 b	15.94 ± 1.67 b	163.39 ± 28.08 b	10.20 ± 0.66 ab
	N2	14.40 ± 0.73 a	168.00 ± 5.18 a	11.68 ± 0.25 a	15.53 ± 1.45 b	179.70 ± 17.90 ab	11.57 ± 0.40 a
	Mean	15.06	166.01	11.05	17.89	185.20	10.47
Source of variation							
Water (W)		*	ns	ns	**	**	ns
Nitrogen (N)		*	ns	**	**	**	**
W * N		*	ns	ns	ns	ns	ns

Note: Values within a column followed by the same letters do not differ significantly (*p* ≤ 0.05) according to LSD tests. W, water addition. N, nitrogen addition. ns, not significant. * significant at *p* < 0.05. ** significant at *p* < 0.01.

**Table 7:** Distribution characteristics of C, N, and P content and stoichiometric ratios in roots, stems, and leaves at seedling stage.

Element	Organ	Min (g kg^−1^)	Max (g kg^−1^)	Mean (g kg^−1^)	SD (g kg^−1^)	CV (/%)
C	Root	397.06	493.34	419.80	107.41	25.59
	Stem	395.73	522.56	416.30	108.02	25.95
	leaf	414.99	525.21	443.16	114.49	25.83
N	Root	2.44	19.23	15.04	3.41	22.66
	Stem	5.14	37.45	13.77	7.19	52.25
	leaf	4.15	31.90	25.83	6.36	24.63
P	Root	0.47	19.50	2.86	3.05	106.76
	Stem	0.48	19.27	2.91	3.01	103.16
	leaf	0.33	12.94	2.78	1.90	68.50
C/N	Root	5.26	40.19	28.68	7.15	24.93
	Stem	18.42	81.20	40.91	20.43	49.95
	leaf	3.90	29.52	17.99	5.19	28.86
C/P	Root	23.13	310.99	186.74	63.25	33.87
	Stem	22.39	277.96	178.84	59.57	33.31
	leaf	16.70	269.57	181.63	53.78	29.61
N/P	Root	1.50	22.18	7.01	3.33	47.53
	Stem	1.62	31.94	6.09	5.08	83.40
	leaf	0.91	12.22	10.31	2.00	19.37

Note: SD, Standard deviation; CV, Coefficient of variation.

**Table 8:** Physical and chemical properties of experimental soil.

PH	SOM (g kg^−1^)	SW (%)	TN (g kg^−1^)	TP (g kg^−1^)	TK (g kg^−1^)	HN (mg kg^−1^)	AP (mg kg^−1^)	AK (mg kg^−1^)
7.2	12.37	8.97	1.63	0.91	11.13	95.3	12.4	112.2

Note: SOM, soil organic matter; SW, soil water content; TN, total N content; TP, total phosphorus; TK, total potassium; HN, hydrolyzed nitrogen; AP, available phosphorous; AK, available potassium content.

## Data Availability

The original contributions presented in the study are included in the article. Further inquiries can be directed to the corresponding author.
